# *In vitro* antioxidant capacities of eight different kinds of apples and their effects on lipopolysaccharide-induced oxidative damage in mice

**DOI:** 10.1371/journal.pone.0191762

**Published:** 2018-01-25

**Authors:** Shuang Guo, Yuehua Wang, Shurui Chou, Huijun Cui, Dongnan Li, Bin Li

**Affiliations:** College of Food Science, Shenyang Agricultural University, Liaoning, China; Jadavpur University, INDIA

## Abstract

In the present study, the DPPH and ABTS^+^ radical scavenging activity of eight types of apples decreased (*P* < 0.05) during the 70-day storage at 4°C. The Fushi (F2) apples from Xin Jiang showed the highest radical scavenging activity. For *in vivo* study, 40 male Kunming mice (body weight 20–25 g) were selected and randomly assigned to four groups (10 mice per group). The F2 groups (F2S, F2 + sterile saline and F2L, F2 + lipopolysaccharide) were administered with 0.3 mL F2 filtrate via gastric intubation daily for 28 days. The control groups (CS, CON + sterile saline and CL, CON + lipopolysaccharide) were treated with sterile saline at the same volume. At day 29, mice of F2L and CL groups were injected with 100 μg/kg body weight of lipopolysaccharide (LPS) intraperitoneally, while those of F2S and CS groups were injected equal volume of sterile saline. In comparison to the CS group, the CL group showed a decrease (*P* < 0.05) in serum, liver, and hepatic mitochondrial antioxidant capacity, reduction (*P* < 0.05) in the expression of hepatic antioxidant-related genes, and an increase (*P* < 0.05) in serum alanine aminotransferase (ALT), aspartate aminotransferase (AST), malondialdehyde (MDA), protein carbonyl (PC), and reactive oxygen species (ROS). In comparison to the CL group, the F2L group showed lower (*P* < 0.05) levels of serum ALT, AST, and ROS, higher (*P* < 0.05) level of serum, liver, and hepatic mitochondrial antioxidant capacity, increased mitochondrial membrane potential (MMP), and enhanced (*P* < 0.05) expression of hepatic antioxidant-related genes. These results suggest that F2 may exert protective effect against LPS-induced oxidative damage by improving the antioxidant capacity.

## Introduction

Lipid peroxidation may contribute to the development of oxygen radical-related injury and is one of the important causes of cell membrane damages [1]. Oxidative damage is induced upon disturbance of the balance between the antioxidant system and free radical generation system, leading to several diseases. Previous studies have associated excessive free radicals with neuronal disorders [2]. Lipopolysaccharide (LPS), a cell wall component of gram-negative bacteria, causes severe inflammation, septic shock, and systemic inflammatory response syndrome [3]. Oxidative damage induced by LPS injection results in the overproduction of free radicals, including reactive-oxygen species (ROS) [4]. LPS has been suggested to be very useful for the study of oxidative damage in laboratory animals [5,6], which was thought to induce the hepatic damage [7,8].

Fruit consumption is important to maintain health and may reduce the risk of diseases. Most fruits contain necessary nutrients, fiber, antioxidants, phytochemicals, and other bioactive compounds. The antioxidant present in fruits mitigates the consequences of oxidative damage associated with chronic disease development and ageing [9]. Apple, one of the most frequently consumed fruit, is famous for its high contents of beneficial compounds such fiber, minerals, antioxidants, and other biologically active molecules [10]. The antioxidant activity of a fruit is related to their antioxidant enzymes structures, which is influenced by many factors, including genetic factors, storage time, and packaging method [11–13]. Previous studies have shown that dietary antioxidants exhibited beneficial effects and improved the quality of life by counteracting the overproduced free radicals. Antioxidant enzymes or natural products may suppress the oxidative damage through their antioxidative function. This study was designed to evaluate the free radicals scavenging capacity of eight different types of apples *in vitro* and evaluate their effects on the LPS-induced oxidative damage in mice.

## Materials and methods

### Apples

Around 30 kg of apples were collected from each of the eight varieties, including Liao Ning (Guoguang, G), Qiaonajin (Q), Shan Dong (Hongfushi, H1), Shan Xi (Podingqinguan, P), Gan Su (Huaniu, H2), Fushi (F1), He Bei (Wanglin, W), and Xin Jiang (Fushi, F2), from China in 2016 and stored at 4°C in the dark until subsequent analysis. Apples were collected from the same plants and used for the preparation of juices during the 70-day storage at 4°C.

Apple samples were taken every 14 days during the 70 days of storage at 4°C and the samples were cut into six pieces using a sharp knife. The operation was performed for 2 min under water by limiting oxygen diffusion to prevent enzymatic browning. The samples were diluted with sterile saline (1:9, v/v) and blended at 10,000 rpm for 1 min using a homogenizer. The homogenized samples were centrifuged at 2,800 × g for 15 min at 4°C and the supernatants used to analyze the *in vitro* antioxidant activity.

### DPPH assay

DPPH assay was performed according to the standard protocol [14]. DPPH was mixed with ethanol to obtain a 0.1 mM solution and stored in dark. Sample supernatants were mixed with the reagent and shaken vigorously. The solution was incubated in dark at 24°C for 30 min before measuring the absorbance at 517 nm wavelength with a spectrophotometer. DPPH radical scavenging activity was calculated using the following equation:

$$DPPH\;radical\;scavenging\;activity\;\left(\%\right)=\left(A_{control}-A_{sample}\right)/A_{control}\times 100\%$$

where A_control_ was the absorbance of the control and A_sample_ was the absorbance of the sample under same conditions.

### ABTS^+^ assay

We employed the method described by Siddhuraju et al. [15] to perform ABTS^+^ assay. ABTS^+^ working solution was prepared by mixing 7 mM ABTS^+^ stock solution and 2.45 mM potassium persulfate (K_2_S_2_O_8_) solution, followed by incubation in the dark at 24°C for 12–16 h. ABTS^+^ solution was diluted with ethanol before use to achieve an absorbance of 0.70 ± 0.02 at 734 nm wavelength. The assay was performed by incubating 1 mL ABTS^+^ solution with 3 mL sample supernatants for 30 min at 30°C, followed by absorbance measurement at 534 nm wavelength. ABTS^+^ radical scavenging activity was calculated using the following equation:

$$ABTS^{+}\;radical\;scavenging\;activity\;(\%)=\left(A_{control}-A_{sample}\right)/A_{control}\times 100\%$$

where A_control_ was the absorbance of the control and A_sample_ was the absorbance of the sample under same conditions.

### *In vivo* experimental design

For the *in vivo* study, F2 was diluted with sterile saline (1:9, v/v) and blended at 10,000 rpm for 1 min using homogenizer. The homogenized samples were centrifuged at 4,000 rpm for 15 min at 4°C and the supernatant was used for further study. The *in vivo* study was performed using 40 male Kunming mice (body weight [BW] 20–25 g) randomly assigned to four groups (10 mice per group). F2 groups (F2S, F2L) were administered with the F2 supernatant via gastric intubation at a dose of 0.3 mL twice daily for 28 days. The control groups (CS, CL) were treated with sterile saline at the same volume. All mice were fed with common basal diet and no mortality was reported during the intubation. LPS (*Escherichia coli* 0111:B4, purchased from Sigma, USA) was prepared in 0.9% sterile saline. At 29 day, mice of F2L and CL groups were administered with 100 μg/kg BW of LPS intraperitoneally, while mice of F2S and CS groups were administered with 100 μg/kg BW of 0.9% sterile saline.

### Housing of animals

Four groups of mice were raised under controlled conditions with 25 ± 3°C temperature, 60 ± 10% humidity, and a 12/12 light-dark cycle. Mice were provided water and diet ad libitum. The experiment was approved and conducted under the supervision of Animal Care and Use Committee, Shenyang Agricultural University, Liaoning, People's Republic of China. Progressive deterioration of the animals' health leading to death was not allowed. Humane endpoints were set to decide the time to sacrifice mice and were as follows: body temperature and physical activity are significantly worse than those of active mice in a few hours, mice show no response to intermittent stimulation thrice in 30 min, or the respiratory rate of mice is rapid or slow. The workers monitored the health of each mouse every 6 h and strictly performed the rules of humane endpoints. At day 29 of raising, all mice were anesthetized by intraperitoneal injection of 100 mg/kg pentobarbital (Sigma, USA) and sacrificed under the condition of limb paralysis or unable to right themselves in 15 s when placed on their side.

### Determination of serum alanine transaminase (ALT) and aspartate transaminase (AST)

All mice were anesthetized and slaughtered 24 h after LPS injection. Blood was obtained from the sacrificed animals and centrifuged at 3,500 rpm for 15 min at 4°C. The levels of serum alanine aminotransferase (ALT, No. C009) and serum aspartate aminotransferase (AST, No. C010) were determined from triplicate samples using commercial diagnostic kits (Nanjing Jiancheng Institute of Bioengineering, Jiangsu, China).

### Determination of antioxidant system

Mice livers (1 g) were homogenized at 8,000 rpm for 10 s in 9 mL of 0.9% sodium chloride buffer on ice and centrifuged at 4,000 rpm at 4°C for 15 min. The blood samples were centrifuged at 3,500 rpm at 4°C for 15 min. Liver supernatants and serum samples were individually used to measure the activity of superoxide dismutase (SOD, No. A001), glutathione peroxidase (GSH-Px, No. A005), total antioxidant capacity (T-AOC, No. A007), and malondialdehyde (MDA, No. A003) in triplicates using the corresponding diagnostic kits (Nanjing Jiancheng Institute of Bioengineering, Jiangsu, China).

### Isolation of mice liver mitochondria

Hepatic mitochondria were prepared according to the method described by Tang [16]. Briefly, mice livers were homogenized in ice-chilled Dounce homogenizers (1:10, w/v) using an isolation buffer containing 10 mM MOPS pH 7.4, 250 mM sucrose, 5 mM KH_2_PO_4_, 2 mM MgCl_2_, 1 mM EGTA, and 0.1% fatty acid-free bovine serum albumin, followed by centrifugation at 1,000 × *g* for 5 min at 4°C. The supernatant was dispensed and the pellet resuspended and washed with the isolation buffer, followed by centrifugation at 12,000 ×*g* for 5 min. Mitochondria were lysed and the proteins measured using the Micro bicinchoninic acid (BCA, No. A045-3) protein assay kit (Nanjing Jiancheng Institute of Bioengineering, Jiangsu, China) according to the manufacturers’ instructions.

### Detection of mitochondrial antioxidant system

The activities of manganese superoxide dismutase (MnSOD, No. A001-2), glutathione (GSH, No. A005), GSH-Px, and MDA in mice liver mitochondria were measured in triplicates using the corresponding diagnostic kits (Nanjing Jiancheng Institute of Bioengineering, Jiangsu, China).

### Measurement of ROS and protein oxidation

The ROS level in mice livers was detected in triplicates using an ROS assay kit (Nanjing Jiancheng Institute of Bioengineering, Jiangsu, China) according to the manufacturer’s instructions. Results were expressed as the mean DCFH-DA fluorescence intensity of the sample over that of the control. Protein oxidation for mice liver mitochondria was calculated using the concentration of protein carbonyl (PC). The PC concentration was measured using a previously described method [17] and presented in nmol/mg protein.

### Measurement of mitochondrial membrane potential (MMP)

The changes in MMP level in mice livers was detected in triplicates using the MMP assay kit (Beyotime Institute of Biotechnology) according to the manufacturer’s instructions.

### Quantitative real-time polymerase chain reaction (PCR)

Total RNA obtained from mice livers using Trizol Reagent (TaKaRa, Dalian, China) was reverse transcribed using a commercial kit (Perfect Real Time, SYBR^®^ PrimeScript™ TaKaRa, China). The mRNA expression level of specific genes was quantified with real-time PCR using SYBR^®^ *Premix Ex Taq*™ II (Tli RNaseH Plus) on an ABI 7300 Fast Real-Time PCR detection system (Applied Biosystems, USA). The SYBR Green PCR reaction mixture comprised 10 μL SYBR^®^ *Premix Ex Taq* (2×), 0.4 μL of the forward and reverse primers, 0.4 μL of ROX reference dye (50×), 6.8 μL of ddH_2_O, and 2 μL of cDNA template. Each sample was amplified in triplicates. The fold-expression of each gene was calculated according to the 2^−**ΔΔ**Ct^ method [18], with *β-actin* gene as an internal standard. The primer sequences used are given in Table 1.

**Table 1:** Primer sequences used for Real-time PCR assay.

Name^1^	Sequence (5’→3’)^2^	Genbank^3^
***β-Actin***	CTGTCCCTGTATGCCTCTG	NM_007393.3
	ATGTCACGCACGATTTCC	
***Nrf2***	CAGTGCTCCTATGCGTGAA	NM_010902.3
	GCGGCTTGAATGTTTGTC	
***HO-1***	ACAGATGGCGTCACTTCG	NM_010442.2
	TGAGGACCCACTGGAGGA	
***MnSOD***	CCGAGGAGAAGTACCACGAG	NM_013671.3
	GCTTGATAGCCTCCAGCAAC	
***GSH-Px***	AGTATGTGTGCTGCTCGGCTCT	NM_008160.6
	CCAGTAATCACCAAGCCAATGC	
***NQO1***	CTTTAGGGTCGTCTTGGC	NM_008706.5
	CAATCAGGGCTCTTCTCG	
***SIRT1***	TGCAGACGTGGTAATGTCCAAAC	NM_019812.2
	ACATCTTGGCAGTATTTGTGGTGAA	
***SIRT3***	TCCGGGAGGTGGGAGAAG	NM_001177804
	ATCCCCTAGCTGGACCACAT	

^1^nuclear factor erythroid 2-related factor 2 (*Nrf2*); heme oxygenase 1 (*HO-1*); manganese superoxide dismutase (*MnSOD*); glutathione peroxidase (*GSH-Px*); NAD(P)H quinone oxidoreductase 1 (*NQO1*); sirtuin 1 (*Sirt1*); sirtuin 3 (*Sirt3*).

^2^Shown as forward primer followed by reverse primer.

^3^ GenBank Accession Number.

### Statistical analysis

All data were subjected to one-way analysis of variance (ANOVA) using the GLM procedure of the Statistical Analysis System (SAS Institute Inc., Cary, NC). When the F test was significant, means were compared using one-factor ANOVA and Bonferroni’s multiple comparison test. Differences between the treatment groups were considered significant at *P* < 0.05. Results are presented as mean ± standard error of the mean (SEM).

## Results

**Fig 1:**
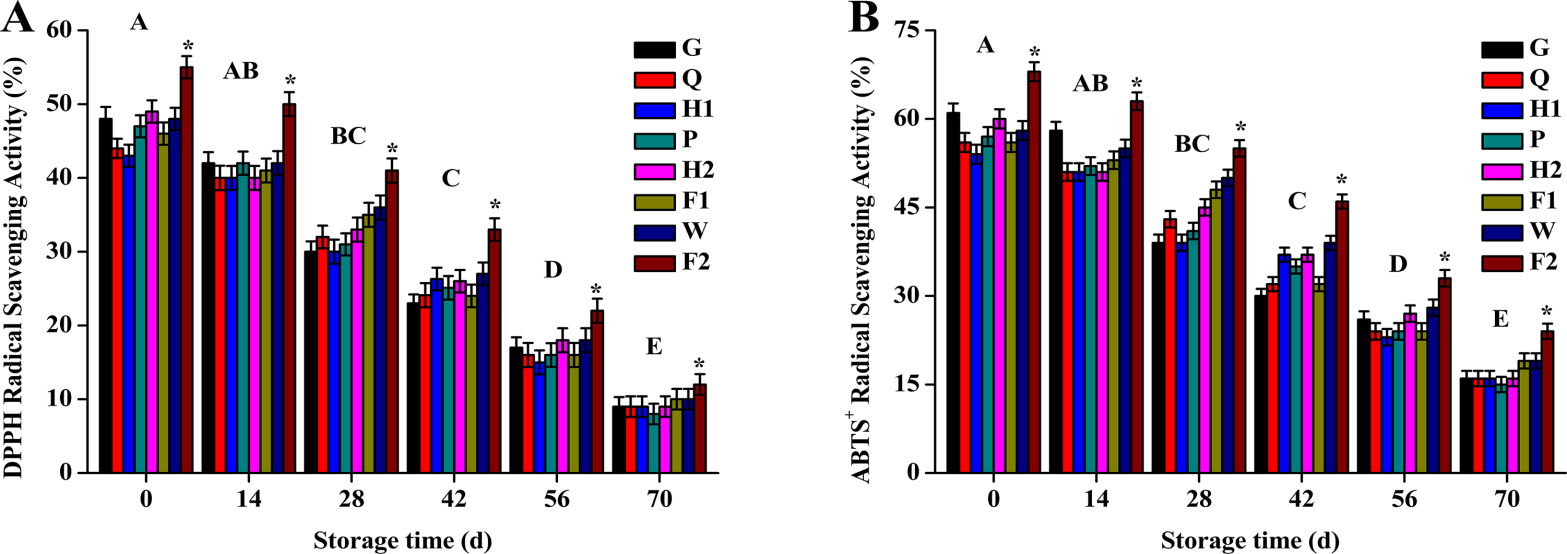
**The DPPH (Fig 1A) and ABTS** ^**+**^ **(Fig 1B) radical scavenging activity of eight different kinds of apples.** Values of DPPH and ABTS^+^ radical scavenging activity are expressed as the mean ± SEM of three independent experiments. 1,1-diphenyl-2-pierylhydrazy (DPPH), 2,2'-Azinobis-(3-ethylbenzthiazoline-6- sulphonate) (ABTS^+^), Liao Ning (Guoguang (G), Qiaonajin (Q)), Shan Dong (Hongfushi (H1)), Shan Xi (Podingqinguan (P)), Gan Su (Huaniu (H2), Fushi (F1)), He Bei (Wanglin (W)), Xin Jiang (Fushi (F2)).

The DPPH and ABTS^+^ radical scavenging activity decreased during the 70-day storage at 4°C. Of the eight apple varieties, F2 apples from Xin Jiang showed the highest (*P* < 0.05, Fig 1) radical scavenging activity during storage.

In comparison to mice in the CL group, those in the F2L group had lower serum ALT and AST levels (*P* < 0.05, Table 2).

**Table 2:** Effects of F2 filtrate on serum ALT and AST level in LPS-injected mice.

	Treatment (n = 10)^1^			
Item^2^	CS	CL	F2S	F2L
**ALT (U/L)**	23.25±2.31^c^	35.61±2.08^a^	18.08±1.01^c^	29.22±2.44^b^
**AST(U/L)**	60.38±1.57^c^	90.01±3.15^a^	52.77±1.87^c^	79.19±2.35^b^

^a-c^ Means in the same row with different superscripts differ (*P* < 0.05).

^1^ CS, gastric intubation in mice with 0.3 ml sterile saline for 28 days and intraperitoneal injection with 100 μg/kg BW of sterile saline at 29 day; CL, gastric intubation in mice with 0.3 ml sterile saline for 28 days and intraperitoneal injection with 100 μg/kg BW of LPS at 29 day; F2S, gastric intubation in mice with 0.3 ml F2 filtrate twice daily for 28 days and intraperitoneal injection with 100 μg/kg BW of sterile saline at 29 day; F2L, gastric intubation in mice with 0.3 ml F2 filtrate twice daily for 28 days and intraperitoneal injection with 100 μg/kg BW of LPS at 29 day.

^2^ alanine aminotransferase (ALT), aspartate aminotransferase (AST).

**Fig 2:**
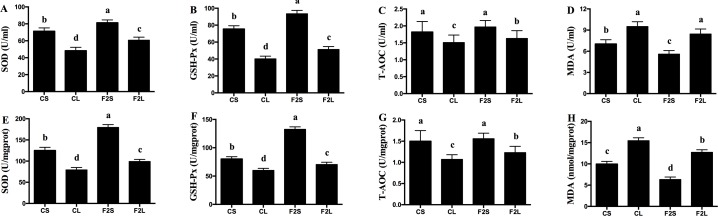
Effects of F2 filtrate on antioxidant capacity in LPS-injected mice. Values are mean ± SEMs (n = 10). Mean of a variable without a common letter differ, *P* < 0.05. (A-D, serum; E-H, liver). CS, gastric intubation in mice with 0.3 ml sterile saline for 28 days and intraperitoneal injection with 100 μg/kg BW of sterile saline at 29 day; CL, gastric intubation in mice with 0.3 ml sterile saline for 28 days and intraperitoneal injection with 100 μg/kg BW of LPS at 29 day; F2S, gastric intubation in mice with 0.3 ml F2 filtrate twice daily for 28 days and intraperitoneal injection with 100 μg/kg BW of sterile saline at 29 day; F2L, gastric intubation in mice with 0.3 ml F2 filtrate twice daily for 28 days and intraperitoneal injection with 100 μg/kg BW of LPS at 29 day. Superoxide dismutase (SOD), glutathione peroxidase (GSH-Px), total antioxidant capacity (T-AOC), malondialdehyde (MDA).

**Fig 3:**
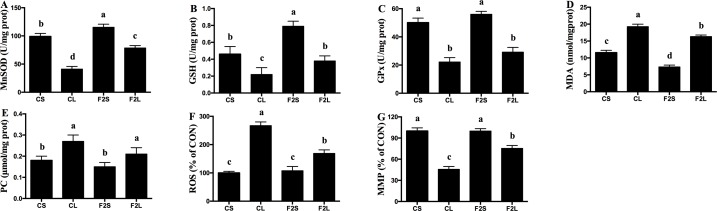
Effects of F2 filtrate on hepatic mitochondrial antioxidant capacity in LPS-injected mice. Values are mean ± SEMs (n = 10). Mean of a variable without a common letter differ, *P* < 0.05. CS, gastric intubation in mice with 0.3 ml sterile saline for 28 days and intraperitoneal injection with 100 μg/kg BW of sterile saline at 29 day; CL, gastric intubation in mice with 0.3 ml sterile saline for 28 days and intraperitoneal injection with 100 μg/kg BW of LPS at 29 day; F2S, gastric intubation in mice with 0.3 ml F2 filtrate twice daily for 28 days and intraperitoneal injection with 100 μg/kg BW of sterile saline at 29 day; F2L, gastric intubation in mice with 0.3 ml F2 filtrate twice daily for 28 days and intraperitoneal injection with 100 μg/kg BW of LPS at 29 day. Manganese superoxide dismutase (MnSOD), glutathione (GSH), glutathione peroxidase (GPx), malondialdehyde (MDA), protein carbonyl (PC), reactive oxygen species (ROS), mitochondrial membrane potential (MMP).

In comparison to the CL group, the F2L group showed an increase (*P* < 0.05) in the serum, liver, and hepatic mitochondrial antioxidant enzyme activity and a decrease in MDA concentrations (*P* < 0.05, Figs 2 and 3). In addition, the F2L group showed lower ROS and PC concentrations (*P* < 0.05) and a higher MMP level (*P* < 0.05, Fig 3) as compared with the CL group.

**Fig 4:**
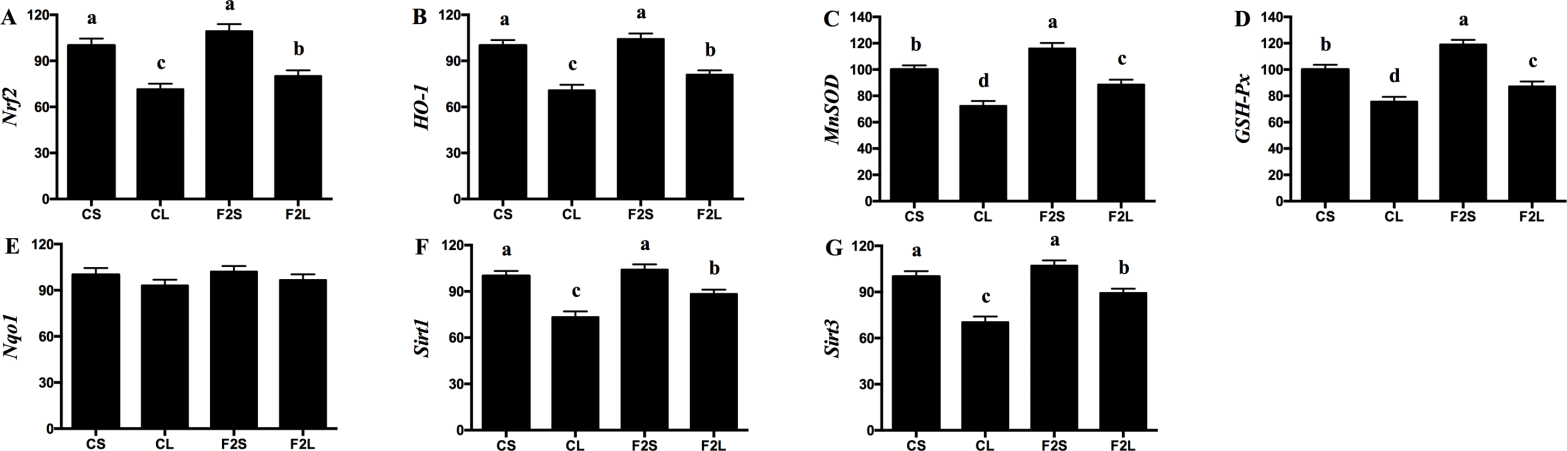
Effects of F2 filtrate on hepatic antioxidant-related genes expression in LPS-injected mice. Values are mean ± SEMs (n = 10). Mean of a variable without a common letter differ, *P* < 0.05. CS, gastric intubation in mice with 0.3 ml sterile saline for 28 days and intraperitoneal injection with 100 μg/kg BW of sterile saline at 29 day; CL, gastric intubation in mice with 0.3 ml sterile saline for 28 days and intraperitoneal injection with 100 μg/kg BW of LPS at 29 day; F2S, gastric intubation in mice with 0.3 ml F2 filtrate twice daily for 28 days and intraperitoneal injection with 100 μg/kg BW of sterile saline at 29 day; F2L, gastric intubation in mice with 0.3 ml F2 filtrate twice daily for 28 days and intraperitoneal injection with 100 μg/kg BW of LPS at 29 day. Nuclear factor erythroid 2-related factor 2 (*Nrf2*), heme oxygenase 1 (*HO-1*), manganese superoxide dismutase (*MnSOD*), glutathione peroxidase 1 (*GSH-Px*), NAD(P)H quinone oxidoreductase 1 (*NQO1*), sirtuin 1 (*Sirt1*), sirtuin 3 (*Sirt3*).

The expression levels of *Nrf2*, *HO-1*, *MnSOD*, *GSH-Px*, *Sirt1*, and *Sirt3* were higher (*P* < 0.05) in the F2L group than the CL group (Fig 4).

## Discussion

Oxidative damage plays an important role during storage of fruits at low or freezing temperatures and may reduce the quality of fruits. Fruits exhibit an array of antioxidant system to overcome the oxidative damage. The radical scavenging activity of DPPH is a standard and easy colorimetric method that has been widely used to calculate the inhibition capacity of free radicals and is based on rapid color changes [19,20]. DPPH measures the efficiency of antioxidants to avoid deterioration of molecules from heat. The structural conformation of the antioxidant determines its mechanical interaction with DPPH [21]. The radical scavenging activity may be influenced by several factors. Hence, ABTS^+^ radical scavenging activity is an alternative assay for the evaluation of the total antioxidant activity of a compound [22]. ABTS^+^ generates a metastable radical with a blue-green color upon oxidation [23]. In the current study, the free radical scavenging activity of eight apples decreased during the 70-day storage at 4°C. Apples from the F2 variety showed the highest free radical scavenging capacity. Agreement with our results, it was reported that some apples exerted strong free radical scavenging capacity [24,25]. Although the values of DPPH and ABTS^+^ assays were similar, we suggest that the F2 filtrate reacted more actively with the ABTS^+^ solution than the DPPH solution.

The levels of ALT and AST in mitochondria and cytoplasm are associated with cell damage and rupture. Hepatic damage is often accompanied with an increase in levels of serum ALT and AST, which are considered as toxicity markers [26]. In the present study, treatment with F2 filtrate could improve the level of serum ALT and AST in mice. These results are in line with a previous study, wherein dietary antioxidants effectively reduced CCl_4_-induced oxidative damage and decreased serum ALT level [27]. In addition, administration of an anti-lipid peroxidation molecule was shown to improve serum ALT and AST levels [28].

Dietary natural antioxidants are gaining popularity, owing to their capacities to protect against several diseases. Fruits are the richest source of antioxidants in human diet. Of these, apple is known not only for its high antioxidant content but also for its popularity among the consumers. ROS is a major product of oxidative damage and may lead to cell and tissue damage upon overproduction. One possible mechanism that contributes to oxidative damage is the disruption of redox status [29]. During animal growth, free radicals are generated through enzymatic and non-enzymatic systems, which may reduce the antioxidant capacity and induce oxidative damage. In addition to health-promoting effects, natural antioxidants may reduce lipid and protein oxidation [30]. Antioxidants arise from different sources such as enzymes, large as well as small molecules, and hormones. In addition, foods such as tomato, garlic, ginger, carotenoids, and apple pomace contain many antioxidants such as vitamin C and E. Antioxidant enzymes prevent oxidative damage and reduce ROS level in the body [31]. Antioxidant enzymes, including SOD and GSH-Px, act as the first defense line during the process of ROS inhibition. SOD promotes the generation of O_2_ and hydrogen peroxide (H_2_O_2_) from O_2_^−^, which in turn are decomposed to water by GSH-Px, thereby preventing the formation of OH^−^. GSH-Px usually participates in the scavenging of free radicals and lipid peroxidation [32]. T-AOC may prevent lipid peroxidation by blocking the peroxidation chain, thereby reducing ROS generation. Previous studies reported that apple exerts an antioxidative effect [33–35] and may enhance the antioxidant capacity of animals, which may inhibit the expression of inflammatory gene and impart protection against oxidant-induced cell damage and cytotoxicity [36,37]. In the present study, the F2 filtrate increased the hepatic mitochondrial antioxidant activity in LPS-challenged mice. The ROS defense system in mitochondria includes enzymatic (MnSOD and GPx) and non-enzymatic (GSH) antioxidants. It has been found that apple may increase the activity of antioxidant enzymes and reduce oxidative damage [38,39]. In the current study, antioxidant enzyme activities were consistent with their corresponding gene expressions. The activation of *Nrf2* transcription factor and its nuclear repressor protein *Keap1* is important in the regulation *MnSOD* and *GSH-Px* gene expression [40]. *HO-1* encodes for heme oxygenase, a rate-limiting enzyme from the heme decomposition reaction, which may generate endogenous carbon monoxide, biliverdin, and ferrous ion Fe^2+^ [41]. The gene *Sirt1* and *Sirt3* are crucial in mitochondrial ROS production, and may improve oxidative damage in cells, which in turn influences the expression of mitochondrial-related gene *MnSOD* and *GSH-Px* [42]. Thus, it can be suggested that the F2 filtrate may enhance the antioxidant capacity in mice through increased antioxidant-related enzyme activities and gene expression and elimination of excess of free radicals.

Mitochondria are vulnerable to the enrichment of polyunsaturated fatty acids in their membranes, which generates more than 95% of ROS in the body. Moderate levels of ROS may improve cell proliferation, development, and differentiation; however, excessive ROS may promote oxidative damage and influence the DNA and RNA functions [43]. Oxidative damage induced by excessive free radicals may damage the mitochondrial membrane, resulting in an increase in the concentration of MDA [44]. MDA is one of the final products of phospholipid peroxidation and may damage the cell membrane. Protein carbonyls are generated through the modification of protein molecules upon oxidation and serve as an important indicator of oxidative damage [45]. In the present study, LPS injection increased the level of ROS, MDA, and PC and decreased the level of MMP in mice hepatic mitochondria. Treatment with F2 filtrate may reduce oxidative damage by decreasing the level of hepatic mitochondrial ROS, MDA, and PC and increasing the level of MMP in LPS-injected mice. Similar results have been reported in a previous study, wherein treatment with antioxidants protected the body from oxidative damage by removing the excess of free radicals [46]. Another study showed that antioxidant treatment reduced the LPS-mediated oxidative damage [47]. It is possible that F2 filtrate may scavenge the excess of free radicals, thereby reducing the oxidative damage induced by LPS.

In conclusion, treatment with F2 filtrate may reduce the oxidative damage in LPS-induced mice. This effect may be associated with an enhancement in the endogenous antioxidant capacity as well as improved expression of the antioxidant-related genes. Future studies should elucidate the molecular mechanism involved in the anti-oxidative effects of apples in LPS-induced mice model.

## References

[1] Koc M, Taysi S, Buyukokuroglu ME, Bakan N. Melatonin protects rat liver against irradiation-induced oxidative injury. Journal of Radiation Research, 2003, 44: 211–215. 14646223 10.1269/jrr.44.211

[2] Naito Y, Yoshikawa T, Matsuyama K, Yagi N, Arai M, Nakamura Y, et al Effects of oxygen radical scavengers on the quality of gastric ulcer healing in rats. Journal of clinical gastroenterology, 1994, 21: S82–86. 8774996

[3] Liu D, Shi J, Colina IA, Kakuda Y, Jun XS. The scavenging capacity and synergistic effects of lycopene, vitamin E, vitamin C, and β-carotene mixtures on the DPPH free radical. LWT-Food Science and Technology, 2008, 41: 1344–1349.

[4] Bian K, Murad F. Diversity of endotoxin-induced nitrotyrosine formation in macrophage-endothelium-rich organs. Free Radical Biology and Medicine, 2001, 31: 421–429. 11498275 10.1016/s0891-5849(01)00600-1

[5] Bai K, Xu W, Zhang J, Kou T, Niu Y, Wan X, et al Assessment of free radical scavenging activity of dimethylglycine sodium salt and its role in providing protection against lipopolysaccharide-induced oxidative stress in mice:. Plos One, 2016, 11(5), e0155393 doi: 10.1371/journal.pone.0155393 27171376 10.1371/journal.pone.0155393 PMC4865141

[6] Cadenas S, Rojas C, Barja G. Endotoxin increases oxidative injury to proteins in guinea pig liver: protection by dietary vitamin C. Pharmacology and Toxicology, 1998, 82: 11–18. 9527640 10.1111/j.1600-0773.1998.tb01391.x

[7] Huang HJ, Chen YH, Liang KC, Jheng YS, Jhao JJ, Su MT, et al Exendin-4 protected against cognitive dysfunction in hyperglycemic mice receiving an intrahippocampal lipopolysaccharide injection. Plos One, 2012, 7(7), e39656 doi: 10.1371/journal.pone.0039656 22844396 10.1371/journal.pone.0039656 PMC3402484

[8] Sun S, Guo Y, Zhao G, Zhou X, Li J, Hu J, et al Complement and the alternative pathway play an important role in lps/d-galn-induced fulminant hepatic failure. Plos One, 2011, 6(11), e26838 doi: 10.1371/journal.pone.0026838 22069473 10.1371/journal.pone.0026838 PMC3206060

[9] Hua Z, Rong T. Dietary polyphenols, oxidative stress and antioxidant and anti-inflammatory effects. Current Opinion in Food Science, 2016, 8: 33–42.

[10] Wu J, Gao H, Zhao L, Liao X, Chen F, Wang Z, et al Chemical compositional characterization of some apple cultivars. Food Chemistry, 2007, 103: 88–93.

[11] Queiroz C, Mlm L, Fialho E, Valente-Mesquita VL. Changes in bioactive compounds and antioxidant capacity of fresh-cut cashew apple. Food Research International, 2011, 44: 1459–1462.

[12] Sun J, Liu SF, Zhang CS, Yu LN, Bi J, Zhu F, et al Chemical composition and antioxidant activities of broussonetia papyrifera fruits. Plos One, 2012, 7(2), e32021 doi: 10.1371/journal.pone.0032021 22389678 10.1371/journal.pone.0032021 PMC3289642

[13] Amakura Y, Umino Y, Sumiko Tsuji A, Tonogai Y. Influence of jam processing on the radical scavenging activity and phenolic content in berries. Journal of Agricultural and Food Chemistry, 2000, 48: 6292–6297. 11312801 10.1021/jf000849z

[14] Moon JK, Shibamoto T. Antioxidant assays for plant and food components. Journal of Agricultural and Food Chemistry, 2009, 57: 1655–1666. doi: 10.1021/jf803537k 19182948 10.1021/jf803537k

[15] Siddhuraju P, Manian S. The antioxidant activity and free radical-scavenging capacity of dietary phenolic extracts from horse gram (Macrotyloma uniflorum (Lam.) Verdc.) seeds. Food Chemistry, 2007, 105: 950–958.

[16] Tang X, Jing G, Wang Y, Fan YM, Xu LZ, Zhao XN, et al Effective protection of Terminalia catappa L. leaves from damage induced by carbon tetrachloride in liver mitochondria. Journal of Nutritional Biochemistry, 2006, 17: 177–182. doi: 10.1016/j.jnutbio.2005.06.008 16169207 10.1016/j.jnutbio.2005.06.008

[17] Wei QY, Chen WF, Bo Z, Li Y, Liu ZL. Inhibition of lipid peroxidation and protein oxidation in rat liver mitochondria by curcumin and its analogues. Biochimica et Biophysica Acta General Subjects, 2006, 1760: 70–77. 10.1016/j.bbagen.2005.09.008 16236451

[18] Liu J, Chen D, Yao Y, Yu B, Mao X, He J, et al Intrauterine growth retardation increases the susceptibility of pigs to high-fat diet-induced mitochondrial dysfunction in skeletal muscle. Plos One, 2012, 7. 10.1371/journal.pone.0034835 PMC3327708 22523560

[19] Mishra K, Ojha H, Chaudhury NK. Estimation of antiradical properties of antioxidants using DPPH assay: A critical review and results. Food Chemistry, 2012, 130: 1036–1043.

[20] Chung HK, Choi CS, Park WJ, Kang MH. Radical scavenging activity of grape-seed extracts prepared from different solvents. Food Science and Biotechnology, 2005, 14: 715–721.

[21] Bondet V, Brandwilliams W, Berset C. Kinetics and mechanisms of antioxidant activity using the DPPH. free radical method. LWT—Food Science and Technology, 1997, 30: 609–615.

[22] Jisang K, Youngsoon L. Antioxidant activity of Maillard reaction products derived from aqueous glucose/glycine, diglycine, and triglycine model systems as a function of heating time. Food Chemistry, 2009, 116: 227–232.

[23] Re R, Pellegrini N, Proteggente A, Pannala A, Yang M, Rice EC. Antioxidant activity applying an improved ABTS radical cation decolorization assay. Free Radical Biology and Medicine, 1999, 26: 1231–1237. 10381194 10.1016/s0891-5849(98)00315-3

[24] Pajk T, Rezar V, Levart A, Salobir J. Efficiency of apples, strawberries, and tomatoes for reduction of oxidative stress in pigs as a model for humans. Nutrition, 2006, 22: 376–384. doi: 10.1016/j.nut.2005.08.010 16413749 10.1016/j.nut.2005.08.010

[25] Skupień K, Kostrzewanowak D, Oszmiański J, Tarasiuk J. In vitro antileukaemic activity of extracts from chokeberry (Aronia melanocarpa [Michx] Elliott) and mulberry (Morus alba L.) leaves against sensitive and multidrug resistant HL60 cells. Phytotherapy Research, 2008, 22: 689–694. doi: 10.1002/ptr.2411 18350513 10.1002/ptr.2411

[26] Ramesh B, Karuna R, Reddy SS, Haritha K, Mangala SD, Sasi BR, et al Effect of Commiphora mukul gum resin on hepatic marker enzymes, lipid peroxidation and antioxidants status in pancreas and heart of streptozotocin induced diabetic rats. Asian Pacific Journal of Tropical Biomedicine, 2012, 2: 895–900. doi: 10.1016/S2221-1691(12)60249-4 23569867 10.1016/S2221-1691(12)60249-4 PMC3609238

[27] Tipoe GL, Leung TM, Liong EC, Lau TYH, Fung ML, Nanji AA. Epigallocatechin-3-gallate (EGCG) reduces liver inflammation, oxidative stress and fibrosis in carbon tetrachloride (CCl 4)-induced liver injury in mice. Toxicology, 2010, 273: 45–52. doi: 10.1016/j.tox.2010.04.014 20438794 10.1016/j.tox.2010.04.014

[28] Hsu DZ, Liu MY. Sesame oil protects against lipopolysaccharide- stimulated oxidative stress in rats. Critical care medicine, 2004, 32: 227–231. doi: 10.1097/01.CCM.0000104947.16669.29 14707583 10.1097/01.CCM.0000104947.16669.29

[29] Ravikumar V, Shivashangari KS, Devaki T. Effect of tridax procumbens on liver antioxidant defense system during lipopolysaccharide-induced hepatitis in D-galactosamine sensitised rats. Molecular and cellular biochemistry, 2005, 269: 131–136. 15786725 10.1007/s11010-005-3443-z

[30] Halliwell B. Antioxidants in human health and disease. Nutrition, 1996, 16: 33–50. 10.1146/annurev.nu.16.070196.000341 8839918

[31] Wang J, Zhang W, Sun D, Song L, Li Y, Xu C. Analysis of neuroglobin mRNA expression in rat brain due to arsenite‐induced oxidative stress. Environmental toxicology, 2012, 27: 503–509. doi: 10.1002/tox.20664 22887765 10.1002/tox.20664

[32] Shirazi A, Mihandoost E, Ghobadi G, Mohseni M, Ghazi-Khansari M. Evaluation of radio-protective effect of melatonin on whole body irradiation induced liver tissue damage. Cell Journal, 2013, 14: 292–297. 23577309 PMC3593934

[33] K WL, Y JK, Kim DO, And HJL, Yong LC. Major phenolics in apple and their contribution to the total antioxidant capacity. Journal of Agricultural and Food Chemistry, 2003, 51: 6516–6520. doi: 10.1021/jf034475w 14558772 10.1021/jf034475w

[34] Tsao R, Yang R, Xie S, Sockovie E, Khanizadeh S. Which polyphenolic compounds contribute to the total antioxidant activities of apple? Journal of Agricultural and Food Chemistry, 2005, 53: 4989–4995. doi: 10.1021/jf048289h 15941346 10.1021/jf048289h

[35] Wojdyło A, Oszmiański J, Laskowski P. Polyphenolic compounds and antioxidant activity of new and old apple varieties. Journal of Agricultural and Food Chemistry, 2008, 56: 6520–6530. doi: 10.1021/jf800510j 18611028 10.1021/jf800510j

[36] Bonarska-Kujawa D, Pruchnik H, Oszmiański J, Sarapuk J, Kleszczyńska H. Changes caused by fruit extracts in the lipid phase of biological and model membranes. Food Biophysics, 2011, 6: 58–67. doi: 10.1007/s11483-010-9175-y 21423329 10.1007/s11483-010-9175-y PMC3034913

[37] Naruszewicz M, Laniewska I, Millo B, Dłuzniewski M. Combination therapy of statin with flavonoids rich extract from chokeberry fruits enhanced reduction in cardiovascular risk markers in patients after myocardial infraction (MI). Atherosclerosis, 2007, 194: 179–184. 10.1016/j.atherosclerosis.2006.12.032 17320090

[38] Bonarska-Kujawa D, Cyboran S, Oszmiański J, Kleszczyńska H. Extracts from apple leaves and fruits as effective antioxidants. Journal of Medicinal Plants Research, 2011, 5: 2339–2347.

[39] Banerjee A, Kunwar A, Mishra B, Priyadarsini KI. Concentration dependent antioxidant/pro-oxidant activity of curcumin studies from AAPH induced hemolysis of RBCs. Chemico-Biological Interactions, 2008, 174: 134–139. doi: 10.1016/j.cbi.2008.05.009 18571152 10.1016/j.cbi.2008.05.009

[40] Kode A, Rajendrasozhan S, Caito S, Yang SR, Megson IL, Rahman I. Resveratrol induces glutathione synthesis by activation of Nrf2 and protects against cigarette smoke-mediated oxidative stress in human lung epithelial cells. American Journal of Physiology Lung Cellular and Molecular Physiology, 2008, 294: 478–488. 10.1152/ajplung.00361.2007 18162601

[41] Morse D, Lin L, Choi AMK, Ryter SW. Heme Oxygenase-1, a critical arbitrator of cell death pathways in lung injury and disease. Free Radical Biology and Medicine, 2009, 47: 1–12. doi: 10.1016/j.freeradbiomed.2009.04.007 19362144 10.1016/j.freeradbiomed.2009.04.007 PMC3078523

[42] Hwang JW, Yao H, Caito S, Sundar IK, Rahman I. Redox regulation of SIRT1 in inflammation and cellular senescence. Free Radical Biology and Medicine, 2013, 61: 95–110. doi: 10.1016/j.freeradbiomed.2013.03.015 23542362 10.1016/j.freeradbiomed.2013.03.015 PMC3762912

[43] Gülçin İ, Elmastaş M, Aboul-Enein HY. Antioxidant activity of clove oil–A powerful antioxidant source. Arabian Journal of chemistry, 2012, 5: 489–499.

[44] Chen JJ, Yu BP. Alterations in mitochondrial membrane fluidity by lipid peroxidation products. Free Radical Biology and Medicine, 1994, 17: 411–418. 7835747 10.1016/0891-5849(94)90167-8

[45] Sohal RS, Agarwal S, Dubey A, Orr WC. Protein oxidative damage is associated with life expectancy of houseflies. Proceedings of the National Academy of Sciences, 1993, 90: 7255–7259. 10.1073/pnas.90.15.7255 PMC47115 8346242

[46] Colle D, Arantes LP, Gubert P, da Luz SCA, Athayde ML, Teixeira Rocha JB, et al Antioxidant properties of Taraxacum officinale leaf extract are involved in the protective effect against hepatoxicity induced by acetaminophen in mice. Journal of medicinal food, 2012, 15: 549–556. doi: 10.1089/jmf.2011.0282 22424457 10.1089/jmf.2011.0282

[47] Brun P, Scarpa M, Palù G, Martines D, Castagliuolo I. Oxidative stress-related damage is implicated in LPS-induced activation of hepatic stellate cells. Digestive and Liver Disease, 2008, 40: A122.

